# Nanocellulose-Assisted Thermally Induced Growth of Silver Nanoparticles for Optical Applications

**DOI:** 10.1021/acsami.1c07544

**Published:** 2021-06-07

**Authors:** Calvin J. Brett, Wiebke Ohm, Björn Fricke, Alexandros E. Alexakis, Tim Laarmann, Volker Körstgens, Peter Müller-Buschbaum, L. Daniel Söderberg, Stephan V. Roth

**Affiliations:** † Department of Engineering Mechanics, KTH Royal Institute of Technology, Teknikringen 8, 100 44 Stockholm, Sweden; ‡ Wallenberg Wood Science Center, KTH Royal Institute of Technology, Teknikringen 56-58, 100 44 Stockholm, Sweden; § Deutsches Elektronen-Synchrotron DESY, Ein Forschungszentrum der Helmholtz-Gemeinschaft, Notkestraße 85, 22607 Hamburg, Germany; ∥ Department of Fibre and Polymer Technology, Division of Coating Technology, Teknikringen 56-58, 100 44 Stockholm, Sweden; ⊥ The Hamburg Centre for Ultrafast Imaging CUI, Luruper Chaussee 149, 22761 Hamburg, Germany; # Lehrstuhl für Funktionelle Materialien, Physik-Department, Technische Universität München, James-Franck-Street 1, 85748 Garching, Germany; ¶ Heinz Maier-Leibnitz Zentrum (MLZ), Technische Universität München, Lichtenbergstraße. 1, 85748 Garching, Germany

**Keywords:** cellulose, silver plasmonics, X-ray scattering, thin films, nucleation and growth, nanoparticles

## Abstract

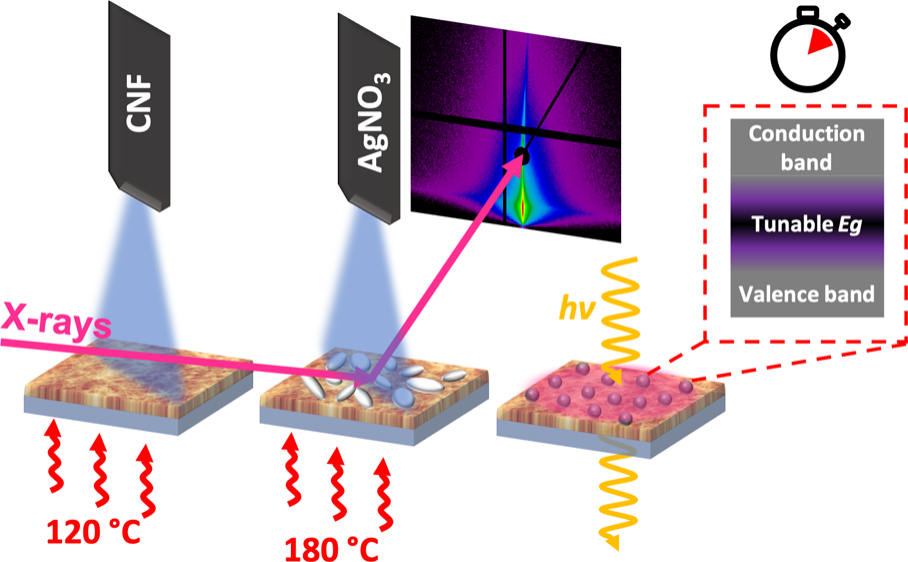

Optically responsive materials are present in everyday life, from screens to sensors. However, fabricating large-area, fossil-free materials for functional biocompatible applications is still a challenge today. Nanocelluloses from various sources, such as wood, can provide biocompatibility and are emerging candidates for templating organic optoelectronics. Silver (Ag) in its nanoscale form shows excellent optical properties. Herein, we combine both materials using thin-film large-area spray-coating to study the fabrication of optical response applications. We characterize the Ag nanoparticle formation by X-ray scattering and UV–vis spectroscopy *in situ* during growth on the nanocellulose template. The morphology and optical properties of the nanocellulose film are compared to the rigid reference surface SiO_2_. Our results clearly show the potential to tailor the energy band gap of the resulting hybrid material.

## 1 Introduction

In our modern world, we are highly dependent on automation and digitalization, often through a man–machine interaction using sensors and devices that react to our input. As sustainability is one of the global challenges, we also need to increase the sustainability of these sensors and devices, ranging from the harvesting of raw materials and recyclability to end-of-life processing, including environmentally friendly processes. Historically, most furniture and much of housing and construction tools have been using wood as the primary raw material. However, wood-based nanocellulose has rarely been considered as a component of smart sensors.^1^ The unique properties of cellulose nanofibers (CNFs) reside in their potential to provide material concepts that have high mechanical strength and stiffness at low weight together with recyclability.^2^ Nanocomposite material concepts, with nanocellulose as one of the main components, were shown to provide functionalities for organic supercapacitors, electrodes, and organic solar cell substrates.^3−5^ The effects of water on these material composites, specifically also related to stimuli-responsive changes by humidity, were studied extensively as they predominantly limit their applications.^6−9^ Today, functionalized nanocellulose is used in several forms ranging from films, aerogels, and dispersions to gels to enable tailored applications.^7,10−15^

Metallic nanoparticles of silver (Ag) or gold are excellent candidates for providing optical functionality due to their optoelectronic properties such as plasmonics.^16^ Plasmons provide an excellent opportunity to couple structural changes in nanostructured materials, specifically on surfaces, by their interaction with electromagnetic fields, which are strongly impacted by the structure–property relation of the surface.^17^ Ag nanowire networks are also widely used to produce transparent electric contacts using different deposition techniques such as spray-coating or 3D-printing.^18,19^ Additionally, Ag nanoparticles show excellent catalytic properties and can be sprayed as films for bio-sensing applications.^20,21^ Thus, the combination of the unique features of nanocelluloses and optically active Ag nanoparticles provide an excellent opportunity for fabricating sustainable sensors for future environmentally friendly devices.

With respect to functionalization of nanocellulosic bionanocomposites using Ag nanoparticles, antimicrobial properties have been shown.^22,23^ For the case of bacteria-based cellulose, Ifuku et al. demonstrated the possibility to reduce AgNO_3_ to Ag nanoparticles in an aqueous state by means of the carboxyl groups on the surface of CNFs.^24^ Furthermore, electrically conductive films were fabricated using Ag nanowires and nanocelluloses for transparent conductive papers.^25,26^

Thin-film fabrication can be performed using various techniques such as spin-coating, dip-coating, and spray-coating, where spray deposition is superior over most other laboratory-scale techniques in terms of scalability and the ability to control the nanostructure by tailoring the droplet sizes.^27,28^ By tuning the deposition parameters, the surface morphology and the crystalline structure can be tailored such that the surface diffusion processes can be neglected, which is crucial for plasmonic materials.^29^

The potential to understand spray-deposited cellulosic materials and to retrieve information on decomposition processes of nanoparticles in thin films has been shown by grazing incidence small-angle X-ray scattering (GISAXS).^7,30,31^ By combining this with wide-angle X-ray scattering (WAXS) on wood-based materials, it is possible to correlate crystallite sizes to nanoscale structures and to extract information on the hierarchical morphology of thin films, covering a wide range of length scales.^32^

In the present work, we focus on the fabrication and morphological evolution of the nanostructure during the layering of nanocellulosic plasmonic thin films as an approach to fabricate sensors. We study the impact of a porous nanocellulose substrate on the growth of Ag nanoparticles from an Ag precursor solution. A reference SiO_2_ surface is used to compare the influence of the substrate on the nanoparticle growth. Surface-sensitive, small-angle, and transmission WAXS techniques are applied to follow the growth of the nanoparticles on the different substrates. Scanning electron microscopy (SEM) and transmission electron microscopy (TEM) are used to sample the real space of the different sample surfaces. In transmission light spectroscopy measurements, the plasmonic behavior of the nanoparticles during the growth is studied. In summary, we elucidate that porous cellulose substrates yield a strong plasmonic absorption signal of the Ag nanoparticles using minimal material consumption. However, one aim is also to show that plasmonic effects can be achieved at minimal material consumption, contributing to lower raw material demand, lower environmental impact, as well as lower production costs.

## 2 Experimental Section

### 2.1 Reagents

The nanocellulose was prepared from chemically bleached wood fibers from a mixture of Norwegian spruce (60%) and Scots pine (40%), both from Domsjö AB, Sweden. The fibers were treated with 2,2,6,6-tetramethylpiperidinyl-1-oxyl (TEMPO)-mediated oxidation reaction. The so-called TEMPO–CNF was prepared according to the procedure reported by Isogai et al. and was from the same batch as in previous publication by Brett et al. except that we centrifuged the dispersion at 4500 rpm for 60 min^7,33^ The surface charge induced by carboxyl groups on the CNFs was 1360 μeq/g. The dispersion used had a CNF concentration of 0.07 wt % in water.

For the Ag precursor, 50 mM of silver nitrate (AgNO_3_, ≥99.0% Sigma-Aldrich Corp.) was dissolved in ethanol (99.7%, Carl Roth) and mixed for 15 min in an ultrasonic bath. The solution was used immediately without further storage.

As substrates, double-sided polished fused silica (SiO_2_, JGS1, MicroChemicals GmbH) with a thickness of 700 μm and a size of (25 × 25) mm^2^ was used. The substrates were cleaned with ethanol, acetone, and isopropanol (all Carl Roth) and subsequently flushed with ultrapure water (18.2 MΩ cm^–1^, Milli-Q). Next, they were further cleaned in a highly oxidizing acid bath (87.5 mL with hydrogen peroxide 30%, 190 mL sulfuric acid 96%, 37.5 mL ultrapure water, and all Carl Roth) at 80 °C for 15 min. After the acid bath, the wafers were washed with ultrapure water and kept in it till use. Before spray deposition, the wafers were dried using a nitrogen flow.

### 2.2 Spray Deposition

The spray deposition on the wafers was performed at the P03 beamline with a beamline compatible setup. The spray device (Compact JAU D555000, Spray Systems) was remotely controlled with magnetic valves and a nitrogen flow for the spray deposition. The sample liquids were attached in a 12 mL glass siphon to the spray device. The spray parameters were 1 bar and 200 mm nozzle to sample distance. The CNF templates were prepared as in our previous publication,^7^ comprising spraying for 0.2 s, waiting for 8 s, and repeating this process 20 times at 100 °C. This preparation resulted in a roughness of 2.5 nm.^7^ AgNO_3_ was deposited by a single spray pulse for 0.2 s at room temperature.

### 2.3 X-ray Scattering

Synchrotron-based X-ray scattering experiments were performed at PETRA III, on the P03/MiNaXs beamline. GISAXS and WAXS experiments were performed to study the nanoscale and the atomic scale, respectively. All X-ray experiments were performed with an X-ray energy of *E* = 13.01 keV and sample-to-detector distances SDD_GISAXS_ = (3471 ± 1) mm and SDD_WAXS_ = (105.9 ± 0.5) mm. The beam size during the measurements was (15 × 34) μm^2^. In GISAXS geometry a Pilatus 1M detector (172 μm^2^ pixel size, Dectris Ltd.) and in WAXS geometry a Pilatus 300k detector (172 μm^2^ pixel size, Dectris Ltd.) were used. The samples were sprayed on the beamline and subsequently measured under GISAXS conditions using an incident angle of α_i_ = 0.4°, well above all critical angles within the thin film to access full film information.^7,34^ The GISAXS samples were placed on a heat stage for the reflectance mode (DHS-1100, Anton Paar), allowing for annealing at the beamline. The samples were measured *ex situ* at room temperature and at the equilibrated temperature of 180 °C after 10 min of applied heat.

The WAXS samples were prepared the same way as the GISAXS samples but were measured under normal incidence in transmission geometry. During the WAXS measurements, the samples were mounted in a remotely controllable transmission heater (metal ceramic heater: HT19R; controller: TC200-EC, both Thorlabs Inc.) and the temperature was increased from room temperature to 180 °C in the following steps: 25, 100, 120, 130, 140, 150, 160, 170, and 180 °C. For both GISAXS and WAXS at each temperature step, the sample was laterally moved to distribute the X-ray dose homogeneously and to check for inhomogeneities on the sample. At each temperature step, the sample was measured at 10 spots for 200 ms, which was then later summed up to gain higher statistics. The recorded X-ray scattering patterns were analyzed using the software packages DPDAK 1.2 and OriginPro 2018.^35^

### 2.4 UV–Vis Spectroscopy

The samples for UV–vis spectroscopy were sprayed just before the measurements. The setup consists of the same heat stage as for the WAXS measurements. However, during the UV–vis measurements, the applied temperature was increased constantly to 180 °C, with a temperature rate of 20 °C/s. Every 10 ms, a full spectrum (200–1025 nm) was recorded. The spectroscopic measurements were conducted using a custom-built setup. The light source is a balanced deuterium tungsten source (210–2500 nm, DH-2000-BAL, Ocean Optics), the spectrometer (200–1025 nm, OCEAN-FX-XR1-ES, Ocean Optics), the fibers (600 μm, QP600-1-SR-BX, Ocean Optics), the collimation lens setup (185–2100 nm, UV fused silica, CVA100-COL, LA4647, both Thorlabs Inc.). The data were evaluated using the software package OceanView 1.6.5 (Ocean Optics).

### 2.5 Scanning Electron Microscopy

Field emission SEM measurements were conducted with a Hitachi S-4800 and a liquid-nitrogen-cooled detector. The acceleration power was 5 kV and the working distance was 3.8 mm.

### 2.6 Transmission Electron Microscopy

TEM measurements were conducted with a Hitachi HT7700 on pre-sprayed copper grids. The acceleration power was 100 kV.

## 3 Results and Discussion

### 3.1 Optical Response

**Figure 1:**
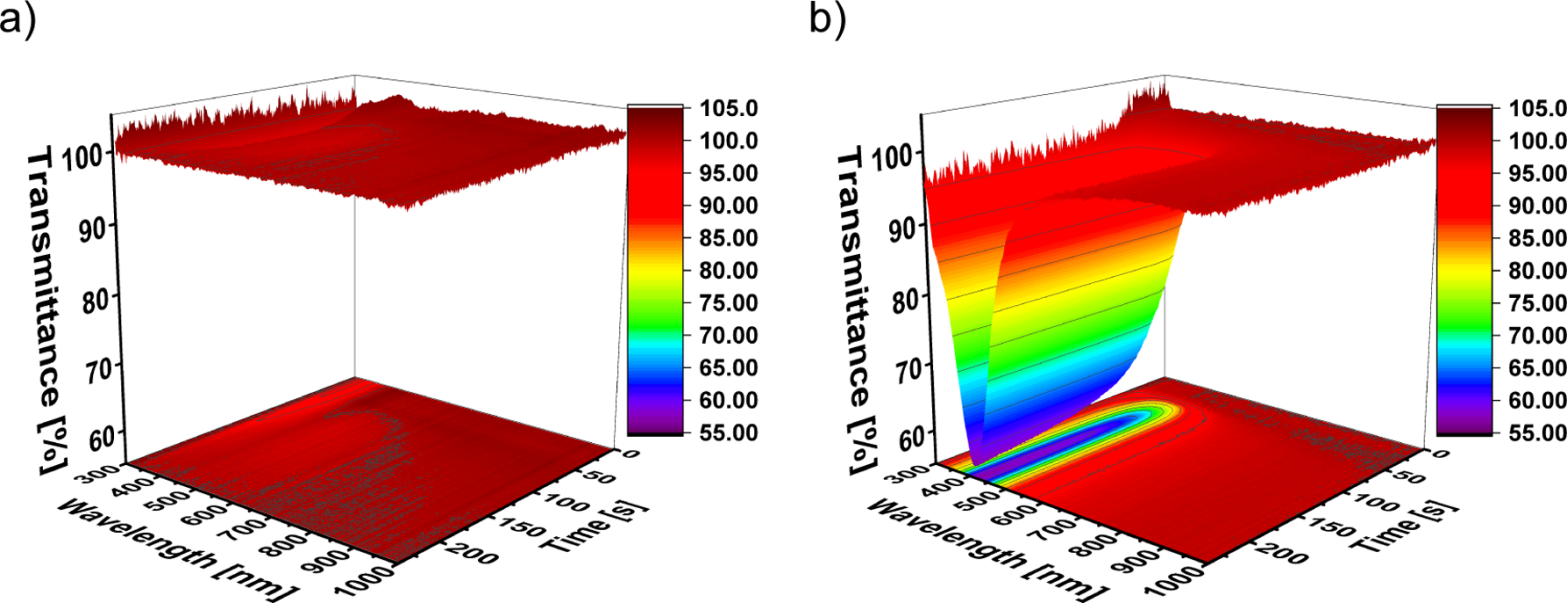
Transmission UV–vis spectroscopic results during the annealing and decomposition of the Ag precursor (a) on silica (SiO_2_) and (b) on a CNF template. The time-dependent transmittance signal is plotted during annealing at *T* = 180 °C.

Two different samples have been prepared for the UV–vis spectroscopy measurements. A single spray pulse of AgNO_3_ solution leading to a sub-monolayer of AgNO_3_ is applied on SiO_2_ and a CNF template (thickness 200 nm). The existing inter- and intramolecular hydrogen bonds within the CNFs formed by hydroxyl groups attract the silver ions directly from the AgNO_3_ when deposited on the CNF template.^36^ The samples are dried for 5 min, mounted in a transmission heater, where the *in situ* transmission UV–vis spectroscopy is performed, see Figure 1. The initial spectra are normalized to the spectrum of the corresponding substrates; hence, only changes occurring during thermal annealing are resolved. In (Figure 1a), the spectra of the AgNO_3_ decomposition on bare SiO_2_ are presented and minor effects can be observed in two regions. One region around 341 nm, where a decline of the absorption peak can be observed, which originates from bare AgNO_3_, see Figure S1. In the second region, between 400 and 500 nm, the opposite trend can be observed, a slight increase in the absorption by 3%, which is an effect of the plasmonic absorption of Ag nanoparticles. This can also be observed for the case of Ag nanoparticle synthesis on a CNF substrate, but with a significantly higher intensity and a lower full-width at half-maximum (fwhm) of the plasmonic peak at (419 ± 1) nm. The smaller-sized Ag nanoparticles on CNFs are an improvement over the large Ag nanoparticles with lower density on SiO_2_. The region around 341 nm is visible during the first 30 s and follows the same trend. The AgNO_3_ peak disappears, at the same time the Ag plasmonic absorption peak appears, see Figure 1b).

**Figure 2:**
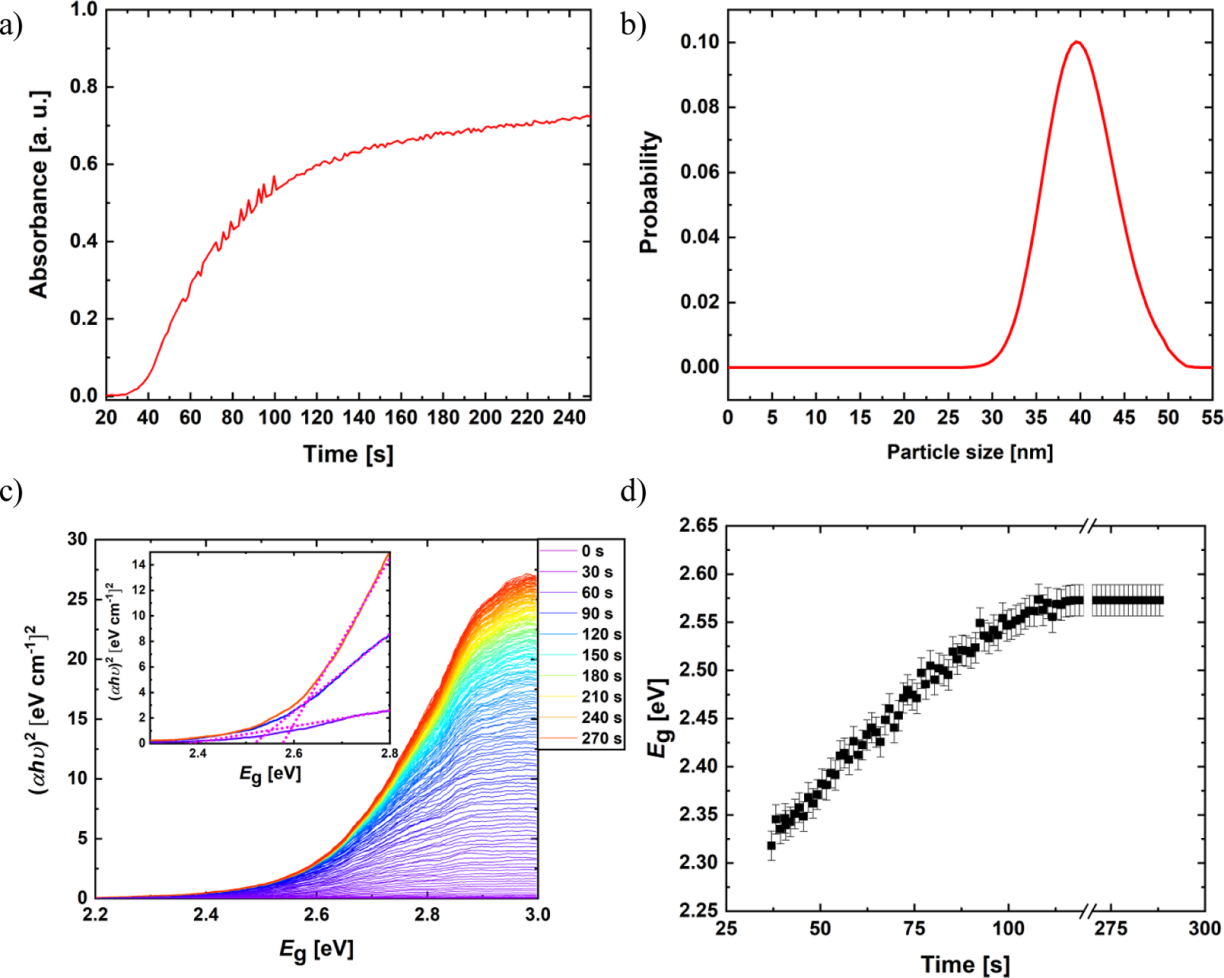
Optical properties extracted from spectroscopic measurements. (a) Peak absorbance of the Ag plasmonic peak. (b) Log–normal distribution of the Ag nanoparticle size on the CNF film at *T* = 180 °C. (c) Tauc plot. The inset in (c) shows the fitting of each curve by a linear fit in the linear region of the peak. (d) Energy band gap extracted from (c).

The low intensity of the silver plasmonic signal on SiO_2_ substrates allowed for an extended analysis only for the case of Ag nanoparticles on a CNF substrate. The deposited amount of AgNO_3_ was equal on both substrates and hence one can conclude already a lower coverage of the Ag nanoparticles after reduction on SiO_2_. The UV–vis spectroscopy data are evaluated using a Mie–Gans fitting approach as introduced by Amendola and Meneghetti for gold nanoparticles in solution.^37^ For the evaluation, the Fermi velocity ν_F_ = 1.39 × 10^6^ m/s and the dielectric function of Ag are fed in the Mathematica program LNMG, where the spectra are fitted, see (Figure 2b).^37,38^ This gives a nanoparticle size of (40.1 ± 3.8) nm with a Log–normal distribution consisting of 98% spherical particles and 2% spheroidal particles, see Figure S1. Liu et al. showed for gold nanoparticles that the electronic band gap is present when the particles are very small, i.e., below 3 nm.^39^

From the UV–vis spectra, we further evaluated the Tauc plot in Figure 2c, where we fit a straight line to the linear part of the peak, see the inset in Figure 2c, to extract the energy band gap of the synthesized Ag nanoparticles, see Figure 2d.^40^ It increases from around *E*_g_ = 2.3 eV to *E*_g_ = 2.58 eV and after 125 s, it remains constant. This feature presents an opportunity for tuning of the energy band gap for optoelectronic applications on a bio-based renewable cellulose substrate. The constant band gap is the metallic silver band gap, meaning that the surrounding medium has not influenced the band gap.

### 3.2 Crystal Size Growth

**Figure 3:**
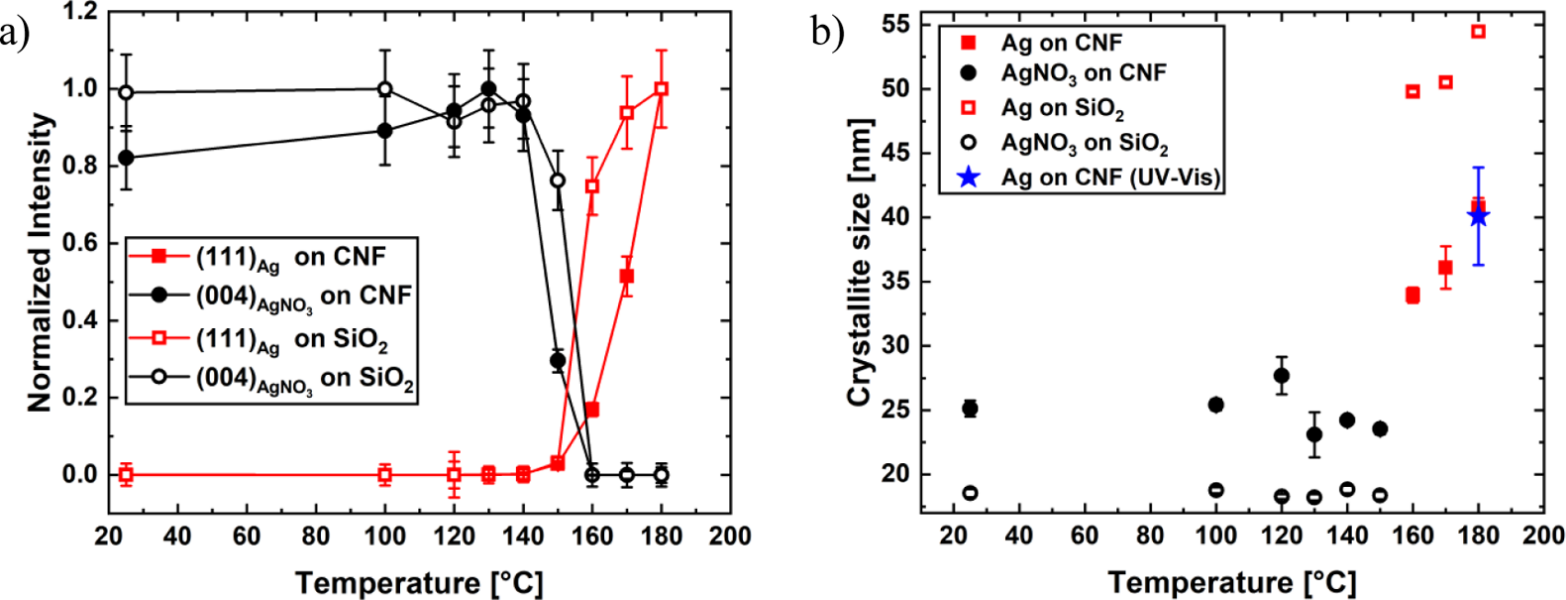
(a) Normalized intensity of WAXS peaks during AgNO_3_ decomposition for the SiO_2_ [open symbols] and CNF substrates [closed symbols]. The intensity of the AgNO_3_(004) [black] and Ag(111) [red] peaks is shown with increasing temperature up to 180 °C. (b) Crystallite sizes calculated using the Scherrer equation and retrieved for AgNO_3_ and metallic Ag nanoparticles. The particle size of the UV–vis [blue] matches with the particle size deduced by WAXS.

WAXS measurements are performed in transmission on the two different substrates. The samples are measured at different temperatures ranging from room temperature up to 180 °C, and the detected AgNO_3_ and Ag Bragg peaks are fitted, see Figure S2. In Figure 3, the normalized intensities of the AgNO_3_(004) and Ag(111) Bragg peaks are plotted *versus* the temperature. The peak intensity changes show that AgNO_3_ disappears entirely between 150 and 160 °C, by transforming into metallic Ag. Carbonization of the CNF template could not be resolved during the thermal annealing. From the Scherrer equation and the fitted fwhm of the peaks, the crystallite sizes are estimated, see Figure 3b. The crystallite size is approximately constant up to 150 °C at (18.5 ± 0.3) nm and (24.8 ± 0.8) nm for AgNO_3_ crystallites on SiO_2_ and CNF substrate, respectively. After transformation to metallic Ag, the nanoparticles grow from (49.7 ± 0.2) to (54.4 ± 0.4) nm on SiO_2_ and from (33.9 ± 0.5) to (40.7 ± 0.7) nm on the CNF substrate. The nanoparticle size estimated from the WAXS data agrees well with the UV–vis result of (40.1 ± 3.8) nm, although with a narrower distribution.

### 3.3 Large-Scale Arrangement

**Figure 4:**
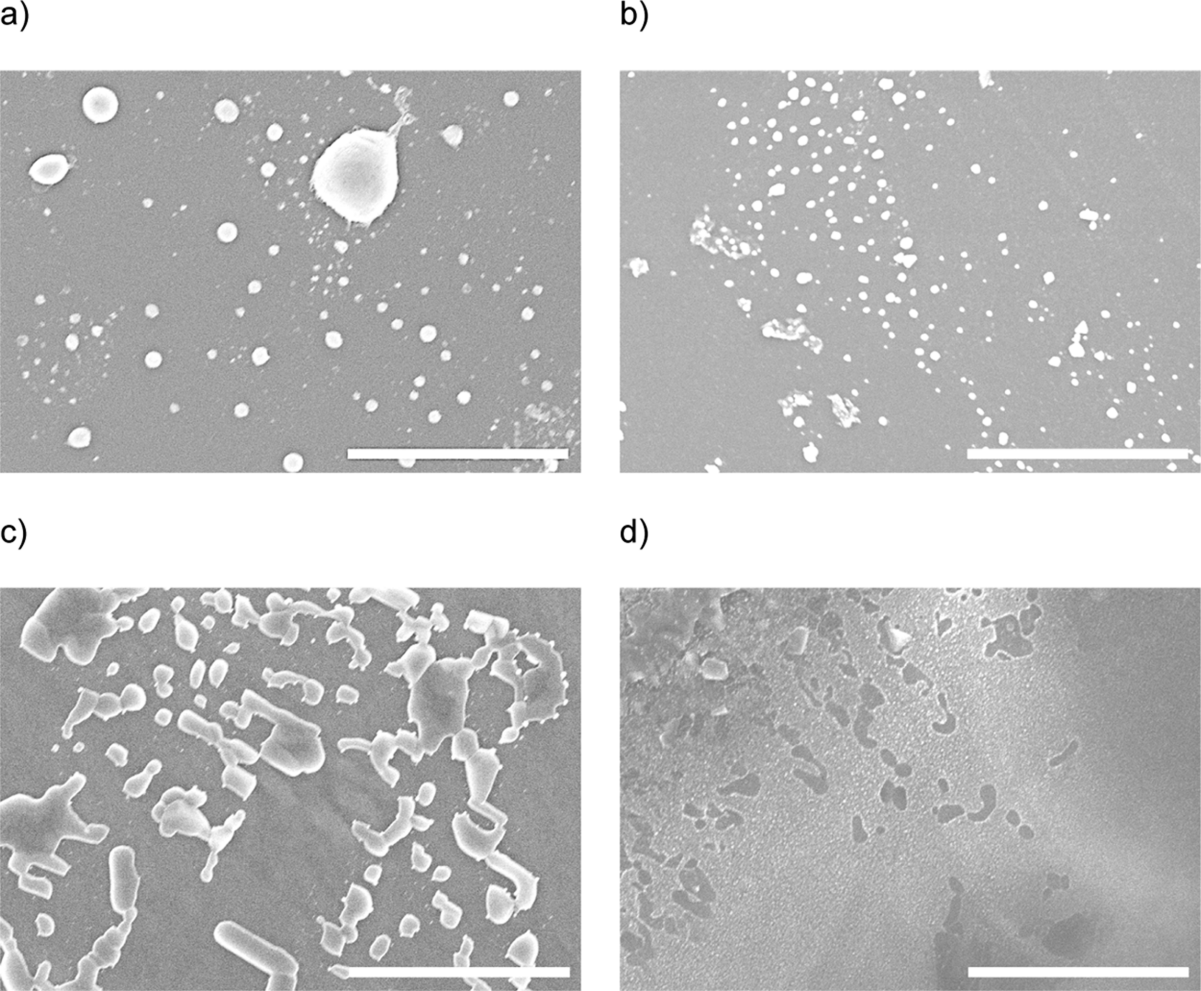
SEM images of the spray-deposited AgNO_3_ precursor on (a,b) SiO_2_ and (c,d) CNF thin films; (a,c) before and (b,d) after annealing to 180 °C. The scale bar equals 4 μm.

SEM measurements are performed on the same samples to gain a real-space image of the spray-deposited AgNO_3_ on the SiO_2_ and the thin CNF templates before and after thermal annealing, see Figure 4. The spray-deposited AgNO_3_ on SiO_2_ in Figure 4a forms visible spherical particles in the few hundreds of nanometer range. On the CNF film seen in Figure 4c, the deposited AgNO_3_ forms more elongated interconnected structures with a wider lateral spread compared to the SiO_2_ substrate, showing a better surface wetting. The shape of the AgNO_3_ on top of the CNFs can be related to the initial rectangular form of the CNF.^41^ In Figure 4b, the thermally annealed AgNO_3_ on SiO_2_ can be seen. One clearly sees that the former macroscopic microscale AgNO_3_ particles decrease in size additionally to the increase mentioned above in the WAXS-retrieved nanoscale nanoparticle size. This is important as the physical properties are governed on the nanoscale. The annealed AgNO_3_ on the cellulose substrate forms a film on top of the cellulose with a granular nanostructure, see Figure 4d.

**Figure 5:**
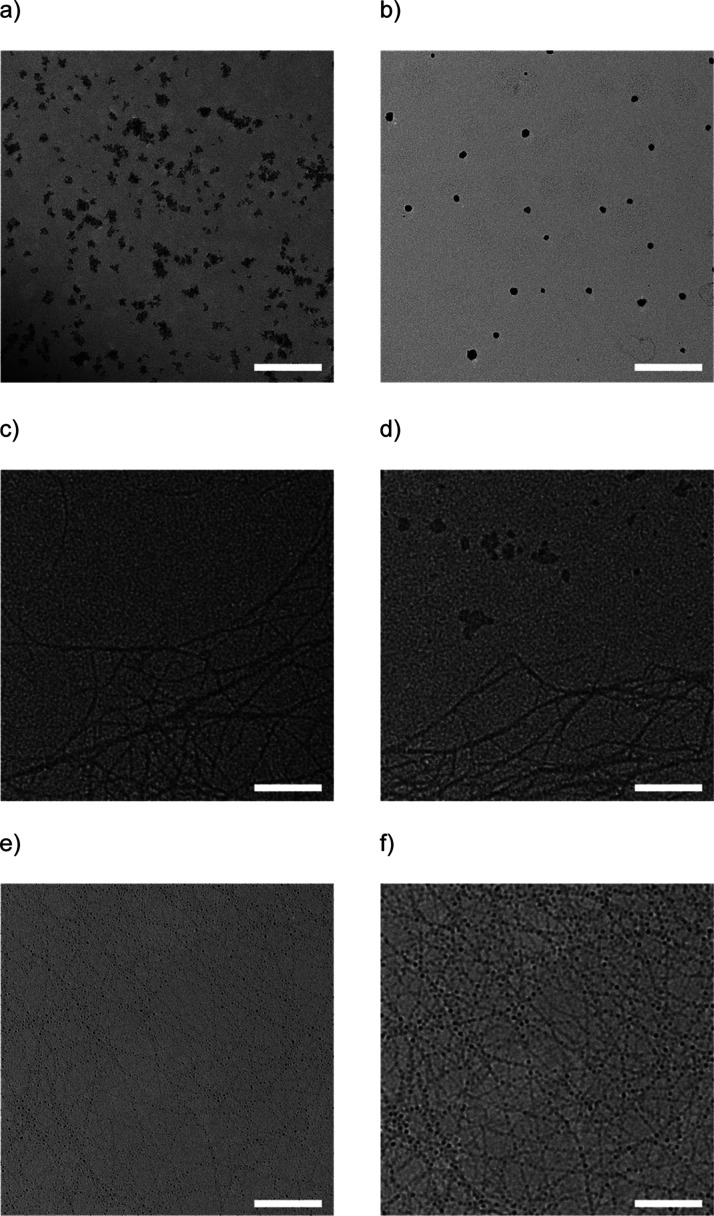
TEM images of the different states of AgNO_3_ and Ag on glass and CNF substrates. (a) AgNO_3_ on glass, (b) Ag on glass, (c) CNFs on glass, (d) AgNO_3_ on CNFs, (e) Ag on CNFs, and (f) Ag on CNFs. Samples (e,f) show the same area with a different focus to visualize the decoration of the CNFs with Ag nanoparticles. The scale bar is 200 nm.

TEM measurements are performed on TEM grids with a single spray pulse of either AgNO_3_, Ag after thermal decomposition or on a single spray pulse of CNFs. In Figure 5a,b, the decomposition of AgNO_3_ to Ag on the SiO_2_ substrate is shown. It is clearly visible that fully separated spheroidal nanoparticles are fabricated. In Figure 5c, the CNF is shown from a single spray pulse, which explains the not fully percolated network. In Figure 5d, the CNF as well as the AgNO_3_ is shown; however, the AgNO_3_ is not visible on the CNF due to lack of contrast. Figure 5e,f shows the CNF network with the Ag nanoparticles after thermal decomposition. Here, the TEM images show the same area with different foci. The nanoparticles seem to decorate the CNF along its fibrous structure and do not appear on the empty spaces in-between the CNF network. The TEM measurements show the guided Ag growth on the CNF agglomerates. We believe that the surface charge induces the localized reduction of the AgNO_3_ and bonding of the growing Ag nanoparticles on these sites. The CNF structure remains intact after thermal annealing to 180 °C, which is well below the thermal degradation temperature of around 220 °C of the TEMPO–CNF.^42^ Previously, the CNF with different surface charges were studied concerning their wettability and morphology.^7,43^ We would expect that a lower surface charge would lead to a lower coverage of the CNF agglomerates.

### 3.4 Nanoscale Self-Assembly

**Figure 6:**
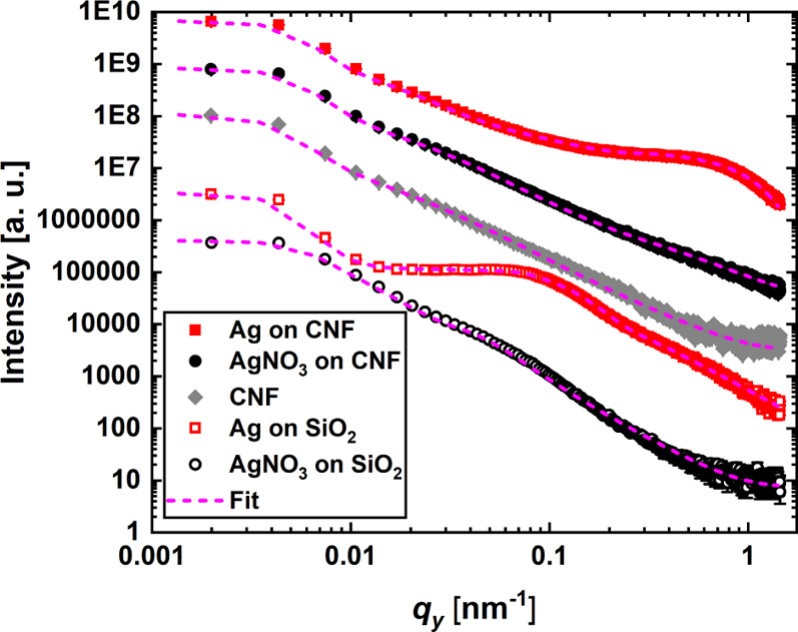
Horizontal line cuts of the 2D GISAXS data (symbols) shown together with fits (dashed lines). The AgNO_3_ samples are shown in black, Ag samples with red symbols, and the CNF with gray symbols. The closed and open symbols represent the sample on CNFs and SiO_2_, respectively. The data are shifted vertically for visualization.

Large-area evaluation of the nanoscale structure of these spray-deposited films is performed with GISAXS, where he samples are prepared as for the UV–vis and WAXS characterization. These samples are annealed *ex situ* and measured directly after thermal treatment. In Figure S3, the scattering patterns of the substrate + AgNO_3_ and substrate + Ag are shown for both substrates. In Figure 6, the horizontal line cuts of the 2D GISAXS data as a function of *q*_*y*_ are shown. The cuts are performed in the Yoneda region of AgNO_3_ and Ag, respectively.

From the scattering patterns as well as from the one-dimensional integrations, one can clearly see the difference for the different substrates. Although the difference is small when depositing AgNO_3_, the transformation to Ag results in significant differences depending on the substrate. Regarding AgNO_3_, structures on the CNF thin film are smaller when compared to the reference SiO_2_ substrate, similar to what is found for macroscopic length scales as seen in Figure 4,5. GISAXS gives a surface-averaged estimate of the mean dimensions of the nanoparticles as well as the structure of their two-dimensional distribution on the surface. After thermal annealing of the AgNO_3_ samples and the full transformation to metallic Ag, we observe an intensity maxima at *q*_*y*_ ≈ 0.1 nm^–1^ and *q*_*y*_ ≈ 0.8 nm^–1^ for the SiO_2_ and CNF substrate, respectively. This implies that we fabricate nanoparticles for both cases, but with distinct size differences: larger nanoparticles on SiO_2_ and smaller ones on CNFs. All fit parameters are reported in Table S1. The CNF substrate structures are similar to the previously published result, where we observe a bimodal cylindrical form factor in a paracrystalline plane.^7^ This model is used and is not changed further to describe the CNF substrate. The deposited AgNO_3_ and the Ag nanoparticles residing on top of the CNF template or the bare SiO_2_ substrate are modeled with either a single or double modal spherical form factor in a paracrystalline plane. On the CNF template, AgNO_3_ decomposes to Ag which causes the nanoparticle radius to change from *r*_AgNO3_ = (7.2 ± 0.4) nm to *r*_Ag_ = (1.9 ± 0.4) nm with a center-to-center distance of the nanoparticles increasing from *d*_AgNO3_ = (40.6 ± 5.6) nm to *d*_Ag_ = (60.2 ± 29.8) nm. Large Ag nanoparticles as extracted from UV–vis and WAXS measurements are not found on the CNF as the size of these particles incidentally overlaps with the initial size of the CNFs. On SiO_2_, AgNO_3_ decomposition results in turning a single-form factor nanostructure into a double-form factor nanostructure. AgNO_3_ nanoparticles have a radius of *r*_AgNO3_ = (37.2 ± 11.5) nm and a center-to-center distance of *d*_AgNO3_ = (95.7 ± 52.2) nm. The large Ag nanoparticles on SiO_2_ have a radius of *r*_1,Ag_ = (27.7 ± 7.7) nm with a center-to-center distance of *d*_1,Ag_ = (255 ± 74) nm and the small ones have a radius of *r*_2,Ag_ = (1.7 ± 0.8) nm with a center-to-center distance of *d*_1,Ag_ = (133 ± 32) nm. We extract from the fitted sizes in GISAXS the surface coverage by calculating the percentage coverage from the individual particles on a square with the size of the center-to-center distance. For AgNO_3_ on the CNF, we have a coverage of ≈9.8%, while Ag on the CNF has a coverage of ≈0.03%. AgNO_3_ on SiO_2_ covers about ≈47.5% and Ag on SiO_2_ has a coverage of ≈3.7% for the large cluster and ≈0.05% for the small cluster. It must be noted that GISAXS resolves the nanoscale objects and larger agglomerates, as seen in SEM, are moved into the resolution limit to enhance the sensitivity toward nanoscale structures, which are difficult to be resolved with SEM. TEM, on the other hand, resolved the nanoparticles to be well distributed and match in sizes with those extracted by GISAXS. Therefore, GISAXS, SEM, and TEM have a certain complementarity. On both substrates, the smaller particles can be found; however, on the bare SiO_2_ substrate, larger structures evolve, which may be attributed to coalescence being inhibited on the porous CNF substrate. Although the same amount of AgNO_3_ is deposited on both substrates, only the CNF substrate exhibits a plasmonic behavior. The loss of a plasmonic signal in the case of the SiO_2_ substrate is attributed to the formation of too large nanostructures on the expense of the plasmon-active small nanostructures, which therefore remain in a too low concentration. The CNF-mediated growth leads to smaller nanoparticles that are also more homogeneously distributed on the surface, enabling a distinct plasmonic behavior.

## 4 Conclusions

We present a facile fabrication of biocompatible thin films with tunable optical responses. By combining spectroscopic methods with X-ray scattering techniques, we are able to elucidate the changes in the nanoparticle dimensions and surface structure distribution before, during, and after the thermal transition of an AgNO_3_ precursor to metallic Ag. The biocompatible substrate is a nanocellulose film, which is compared to a non-porous hard SiO_2_ reference substrate. The CNF template-assisted growth allows us to study, with minimal material usage, the tunability of the electronic band gap by looking into different timescales of the thermal transformation. The soft and nanoporous cellulosic thin films provide a promising route for further evaluation with respect to their use to tune the electronic band gap in applications such as flexible nanoparticle bulk heterojunction solar cells.

## Supporting Information Available

UV–-vis fitting, WAXS data, GISAXS pattern, and GISAXS fit results (PDF)
