# Supplementary material for: Nanocellulose-Assisted Thermally Induced Growth of Silver Nanoparticles for Optical Applications
Source: ACS Appl Mater Interfaces. 2021 Jun 7;13(23):27696–704. doi: 10.1021/acsami.1c07544 (PMC8289233; doi:10.1021/acsami.1c07544)
Supplement: Supplementary file 1 — am1c07544_si_001.pdf [file am1c07544_si_001.pdf]

Supporting Information

Nanocellulose-Assisted Thermally-Induced Growth of Silver Nanoparticles for Optical Applications

Calvin J. Brett[1,2,3], Wiebke Ohm[3], Björn Fricke[3], Alexandros E. Alexakis[2,4], Tim Laarmann[3,5], Volker Körstgens[6], Peter Müller-Buschbaum[6,7], L. Daniel Söderberg[1,2]*, Stephan V. Roth[3,4]**

[1]Department of Engineering Mechanics, KTH Royal Institute of Technology, Teknikringen 8, 100 44 Stockholm, Sweden

[2]Wallenberg Wood Science Center, KTH Royal Institute of Technology, Teknikringen 56-58, 100 44 Stockholm, Sweden

[3]Deutsches Elektronen-Synchrotron DESY, Ein Forschungszentrum der Helmholtz-Gemeinschaft, Notkestraße 85, 22607 Hamburg, Germany

[4]Department of Fibre and Polymer Technology, KTH Royal Institute of Technology, Teknikringen 56-58, 100 44 Stockholm, Sweden

[5]The Hamburg Centre for Ultrafast Imaging CUI, Luruper Chaussee 149, 22761 Hamburg, Germany

[6]Lehrstuhl für Funktionelle Materialien, Physik-Department, Technische Universität München, James-Franck-Str. 1, 85748 Garching, Germany

[7]Heinz Maier-Leibnitz Zentrum (MLZ), Technische Universität München, Lichtenbergstraße. 1, 85748 Garching, Germany

*corresponding author E-Mail: calvinbr@kth.se, dansod@kth.se, stephan.roth@desy.de

co-authors E-Mail: wiebke.ohm@desy.de, bfricke@physnet.uni-hamburg.de, aleale@kth.se, tim.laarmann@desy.de, volker.koerstgens@ph.tum.de, muellerb@ph.tum.de

a) b)

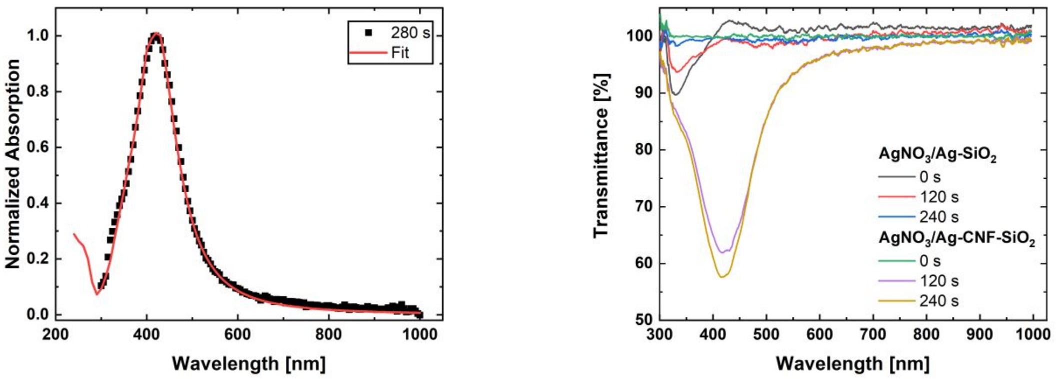

Figure S1. The particle size of the Ag nanoparticles were extracted using the UV-Vis spectroscopy and WAXS measurements. In a) the fit (red line) using the LNMG program is shown for a representative spectrum (raw data at 280 s, black squares). In b) three representative spectra are shown for the two samples at three different time steps (0 s, 120 s, 240 s).

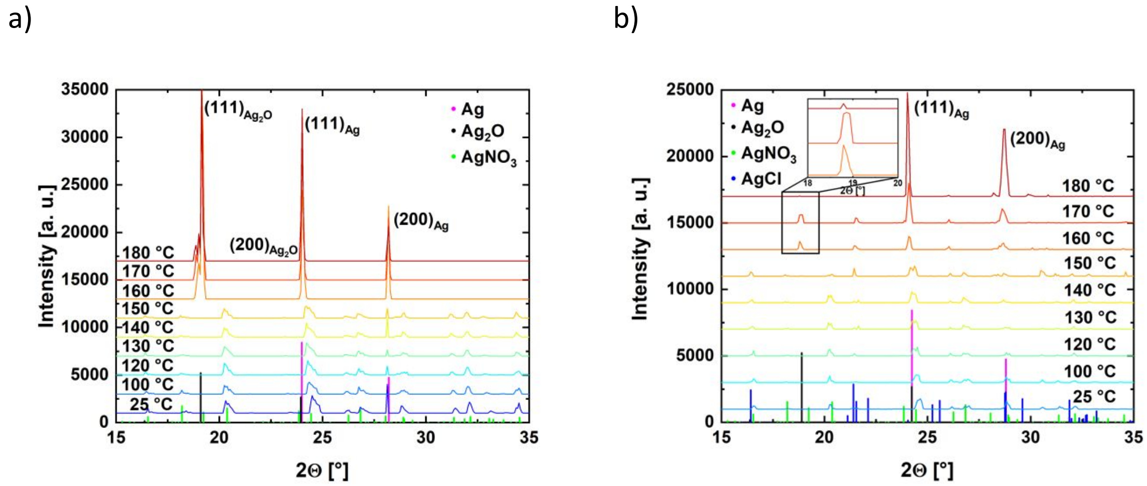

Figure S2. 1D integration of in situ WAXS data during the annealing of the thin films. In a) $AgNO_3$ decompose on SiO_2 and in b) on CNF/SiO_2 respectively to Ag. The inset shows the (111) Ag_2O peak evolution from 160°C to 180°C. The peaks were assigned to the as deposited $AgNO_3$, to silver oxide Ag_2O on SiO_2, AgCl on CNF and pure silver Ag. [40–43]

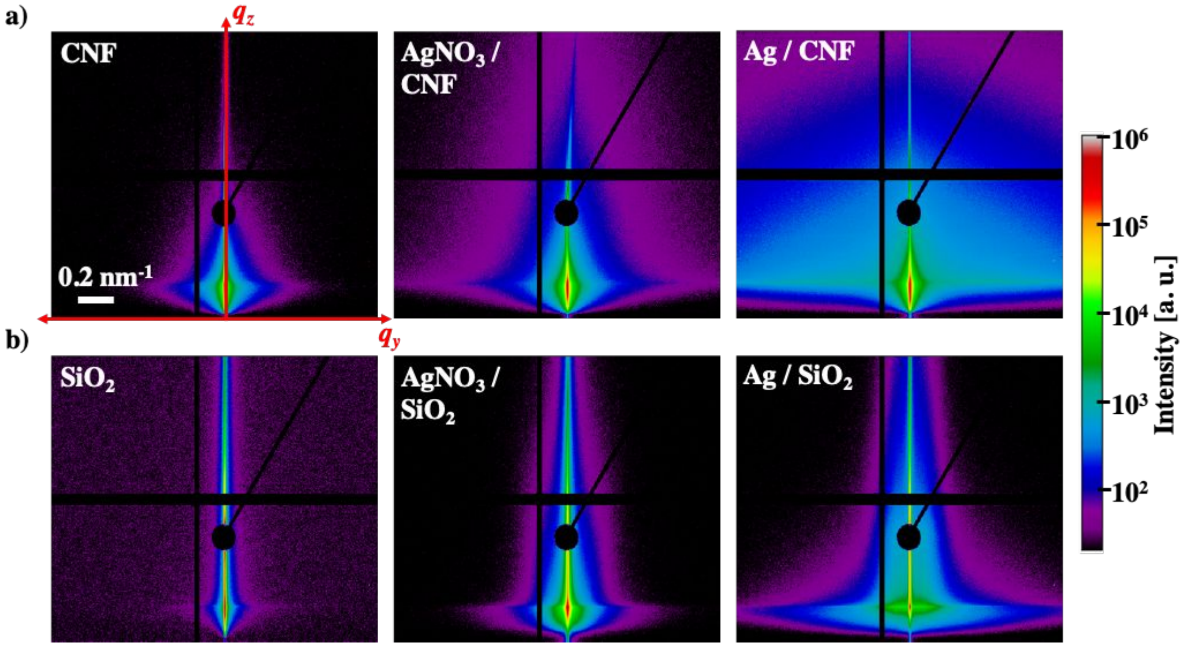

Figure S3. 2D GISAXS data of a) the CNF template, $AgNO_3$ on CNF and Ag on CNF (f.l.t.r) and of b) the SiO_2 substrate, the $AgNO_3$ on SiO_2 and Ag on SiO_2 (f.l.t.r). The beamstop is shielded and black horizontal and vertical lines are the inter detector module gaps.

Table S1. GISAXS fit results retrieved from fitting the one-dimensional horizontal line cuts at the Yoneda region. In the form factor column, the number of used form factors is shown, in case of the samples CNF with $AgNO_3$ and Ag only the data for the full sphere is shown as the substrate CNF is not altered. The shown error estimate is taken from the full width of half maximum of the Gaussian distribution around the average value of the corresponding radii and distances as the typical fit error is only 2%.

	**FF**[a)]	r_1 **[nm]**	d_1 **[nm]**	r_2 **[nm]**	d_2 **[nm]**
**CNF**	2 × c	27.3 ± 12.4	60.9 ± 19.3	6.7 ± 1.4	53.8 ± 4.1
**CNF/$AgNO_3$**	2 × c, 1 × s	7.2 ± 0.4	40.6 ± 5.6		
**CNF/Ag**	2 × c, 1 × s	1.9 ± 0.4	60.2 ± 29.8		
**SiO_2/$AgNO_3$**	1 × s	37.2 ± 11.5	95.7 ± 52.2		
**SiO_2/Ag**	2 × s	27.7 ± 7.7	255 ± 74	1.7 ± 0.8	133 ± 32

[a)]FF = form factor [c = cylinder, s = full sphere]
